# Appropriate use of inhaled corticosteroids in COPD: the candidates for safe withdrawal

**DOI:** 10.1038/npjpcrm.2016.68

**Published:** 2016-09-29

**Authors:** Barbara P Yawn, Samy Suissa, Andrea Rossi

**Affiliations:** 1 Department of Family and Community Health, University of Minnesota, Rochester, MN, USA; 2 Centre for Clinical Epidemiology, Lady Davis Institute, Jewish General Hospital, Montreal, QC, Canada; 3 Department of Epidemiology and Biostatistics and Department of Medicine, McGill University, Montreal, QC, Canada; 4 Pulmonary Unit, University and General Hospital, Verona, Italy

## Abstract

International guidance on chronic obstructive pulmonary disease (COPD) management recommends the use of inhaled corticosteroids (ICS) in those patients at increased likelihood of exacerbation. In spite of this guidance, ICS are prescribed in a large number of patients who are unlikely to benefit. Given the evidence of the risks associated with ICS and the limited indications for their use, there is interest in understanding the effects of withdrawing ICS when prescribed inappropriately. In this review, we discuss the findings of large ICS withdrawal trials, with primary focus on the more recent trials using active comparators. Data from these trials indicate that ICS may be withdrawn without adverse impact on exacerbation risk and patient-reported outcomes in patients with moderate COPD and no history of frequent exacerbations. Considering the safety concerns associated with ICS use, these medications should be withdrawn in patients for whom they are not recommended, while maintaining adequate bronchodilator therapy.

## Introduction

Long-acting bronchodilators are the cornerstone of treatment for chronic obstructive lung disease (COPD) according to the Global Initiative for Chronic Obstructive Lung Disease (GOLD), guidelines from the International Primary Care Respiratory Group and other country-specific organisations.^1–5^ Addition of inhaled corticosteroids (ICS) to regular bronchodilator treatment is recommended for the management of COPD patients with severe-to-very-severe airflow limitation and/or frequent exacerbations (⩾2 per year) not adequately controlled by long-acting bronchodilators, or ⩾1 hospitalisations for exacerbation.^1^ An ICS/long-acting β_2_-agonist (LABA) combination is also appropriate for the management of patients with concomitant asthma and COPD.^1^

Despite recommendations since 2007 limiting ICS use in patients with either forced expiratory volume in 1 s (FEV_1_) <50% or frequent exacerbations, or both,^1,6^ ICS are widely prescribed across all levels of COPD severity and exacerbation risk.^7–14^ In most industrialised countries, it is estimated that ~40% of patients diagnosed with COPD are candidates for ICS according to the GOLD criteria,^15,16^ whereas prescribing rates can exceed 80%.^17^ This frequent prescription of ICS in COPD patients who are unlikely to benefit increases the number of patients at risk of adverse effects, extensively reviewed previously.^18–23^ Therefore, it is worth considering whether and how non-recommended ICS treatment can be withdrawn safely.

Data from an early observational study suggested that ICS withdrawal may lead to rapid occurrence of exacerbations;^24^ however, subsequent real-life and randomised clinical trials obtained different results in patients for whom ICS use is not recommended by guidelines. Here we review the data from recent randomised clinical trials and aim to identify the methods and any risks of withdrawing ICS in patients with COPD. We anticipate this information will help guide primary care physicians and other clinicians in the selection of appropriate patients for whom ICS may be withdrawn or maintained.

## ICS withdrawal trials

Overall, we found five randomised controlled trials with relevant results. The trials varied in design, duration, inclusion criteria, treatment arms, patient number and timing of ICS withdrawal (Table 1).

### Placebo-controlled trials

Only two large placebo-controlled trials were found to include at least some patients with moderate COPD (in our view, those most likely to be candidates for ICS withdrawal).

### COPE

The COPE (COPD study of the department of Pulmonary Medicine, Enschede) study investigated the effect of ICS withdrawal on health status and exacerbations in patients with moderate-to-severe COPD (prebronchodilator FEV_1_ 25–80% predicted).^25^ Exacerbation history and previous ICS use were not considered. Patients received 500 μg of fluticasone propionate (FP) twice daily and 40 μg of ipratropium bromide four times a day for 4 months to optimise lung function; then they were randomised to continue ICS treatment or receive placebo for 6 months. The authors do not disclose whether ipratropium treatment was maintained. Patients in the ICS withdrawal group had earlier exacerbations (hazard ratio (HR) 1.5; 95% confidence interval (CI) 1.05, 2.1) and were more likely to experience rapid recurrent exacerbations (relative risk 4.4; 95% CI 1.9, 10.3) than those in the ICS continuation group. There was also a small, nonsignificant reduction in FEV_1_ (38 ml) and a statistically, but not clinically, significant increase in St George’s Respiratory Questionnaire total score (2.48 units; 95% CI 0.37, 4.58; treatment difference less than the clinical threshold of 4 units^26^) with ICS withdrawal compared with ICS continuation. Serious adverse events were more frequent in the ICS withdrawal group compared with the ICS continuation group. Pneumonia rates were not reported. Unfortunately, no subanalyses were conducted in those with moderate COPD. Study limitations included a relatively short trial duration of 6 months, which may not be long enough to appropriately study deterioration in lung function and exacerbation rate.^25^ In addition, the inclusion criteria took into account exacerbation history only in the month prior to study enrolment.^25^ The lack of information regarding patient exacerbation history limits the conclusions that can be drawn from the study. Although this was the first ICS withdrawal study published in COPD patients, it should be noted that use of ICS monotherapy limits the relevance of these trial data to current clinical practice, as ICS monotherapy is neither approved nor recommended for COPD in many countries.^1^

### WISP

The WISP (Withdrawal of inhaled corticosteroids in people with COPD in primary care) study tested the hypothesis that ICS withdrawal in patients from primary care with COPD who had an FEV_1_>50% predicted and ⩾1 course of antibiotics/oral steroids per year for COPD would result in an increased number of exacerbations, earlier exacerbation onset and a worsening of symptoms.^27^ At baseline, median ICS use was 8 years. After enrolment, existing ICS treatment was withdrawn and patients were randomised to 500 μg of FP twice daily or placebo for one year. Around one-third of patients (31.8% in the ICS withdrawal group and 35.1% in the ICS continuation group) were receiving concurrent LABA therapy during the trial. In the per-protocol analysis, those in the ICS withdrawal group had a higher risk of exacerbation (rate ratio (RR) 1.48; 95% CI 1.17, 1.86; *P*<0.001) and an increase in exacerbation-associated symptoms compared with the ICS continuation group. However, 46% of the ICS withdrawal group returned to their pre-enrolment ICS regimen, compared with 26% of the ICS continuation group. When all study patients were considered (intention-to-treat analysis), exacerbation risk did not differ significantly between the treatment arms. There was no difference in lung function decline or health status between the two groups. The main limitation of this trial was the lack of a consistent approach to exacerbation management and subsequent changes in study inhalers. Post-exacerbation treatment decisions were at the discretion of the general practitioner and patient, resulting in differential study treatment cessation rates and analysis of the data in per-protocol and intention-to-treat populations, in order to reduce potential bias. In addition, the study was designed before current guidelines existed regarding the use of ⩾1 long-acting bronchodilator and the addition of other pharmacological treatments as necessary; this study was, therefore, not conducted in adherence with these guidelines.^1^

### Active comparator trials

Three large trials that included an active comparator are reviewed, and in greater depth than the placebo-controlled trials, as the active comparator makes them more relevant to current COPD management recommendations of long-acting bronchodilator therapy for all patients with moderate-to-very-severe, symptomatic COPD.

### INSTEAD

The objective of INSTEAD (The Indacaterol: Switching Non-exacerbating Patients with Moderate COPD From Salmeterol/Fluticasone to Indacaterol study) was to demonstrate the non-inferiority of indacaterol to LABA/ICS salmeterol/fluticasone propionate (SFC) with respect to trough FEV_1_ at week 12 (with a non-inferiority margin of −60 ml) in patients at low risk for exacerbation, for whom ICS therapy is not currently recommended.^28^ Patients had moderate COPD (FEV_1_ 50–80% predicted) and no exacerbations in the year prior to enrolment. However, all had been prescribed 50/500 μg of SFC twice daily for >3 months prior to enrolment. Patients were randomised 1:1 (*N*=581) to continue SFC treatment or switched to indacaterol monotherapy for a 26-week period.

**Figure 1:**
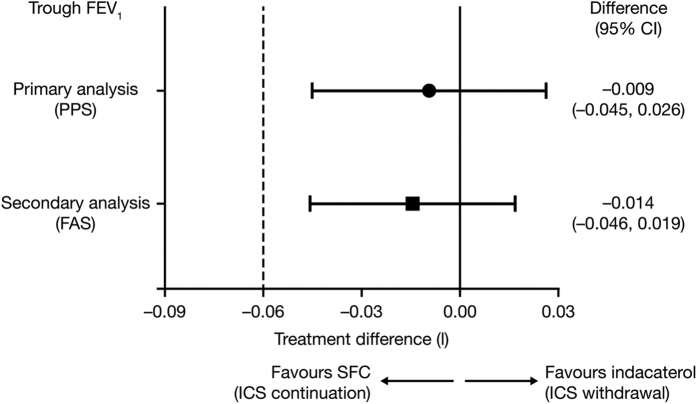
INSTEAD: Non-inferiority of indacaterol to SFC in trough FEV_1_ at week 12 in patients with moderate COPD (FEV_1_ 50–80% predicted) and no exacerbations for >1 year prior to study entry^28^. LSM treatment differences and 95% CI between indacaterol and SFC in trough FEV_1_ at week 12. In non-inferiority testing, the null hypothesis is that the new therapy (here, ICS withdrawal) is inferior to the current therapy (ICS continuation).^59^ This is disproved and non-inferiority established if the efficacy of the new therapy does not exceed the predetermined non-inferiority margin when compared with the current therapy. The dotted line indicates the non-inferiority margin of −60 ml. CI, confidence interval; ICS, inhaled corticosteroid; LSM, least-squares mean; FAS, full analysis set; FEV_1_, forced expiratory volume in 1 s; PPS, per-protocol set (primary analysis); q.d., once daily; SFC, salmeterol/fluticasone propionate. Reproduced with permission of the European Respiratory Society.^60^

**Figure 2:**
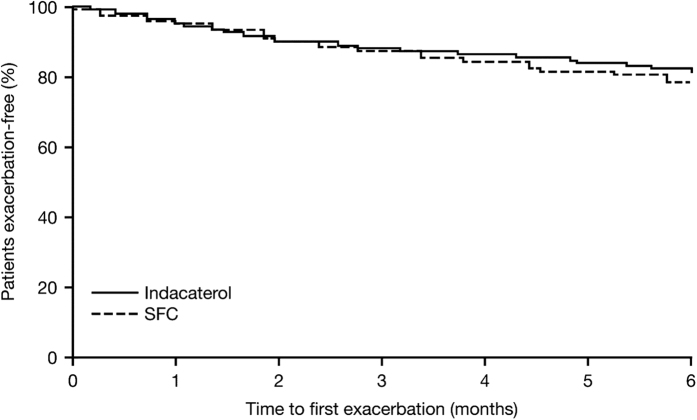
INSTEAD: time to first moderate or severe exacerbation over 26 weeks in patients with moderate COPD (FEV_1_ 50–80% predicted) and no exacerbations for >1 year prior to study entry^28^. SFC, salmeterol/fluticasone propionate. Reproduced with permission of the European Respiratory Society.^60^

Withdrawing patients from SFC to indacaterol monotherapy was non-inferior to SFC continuation for all primary and secondary outcomes, with only −9 ml (95% CI −45, 26) separating the mean trough FEV_1_ after 12 weeks (Figure 1). There was also no significant difference in the rate of any (mild, moderate and severe) exacerbations between the indacaterol and SFC treatment groups (RR 0.86; 95% CI 0.62, 1.20; *P*=0.367), or in the time to first moderate or severe exacerbation in the first 6 months (HR 0.80; *P*=0.258; Figure 2). It should be noted that the study was only 6 months in duration, which is a short follow-up time for exacerbations. However, the authors indicate that any numerical increase in the rate of exacerbations with indacaterol may be interpreted as a signal that exacerbations were being triggered. Also, of note is that the study was powered to assess lung function, not exacerbations, which should be taken into account when making any inference with regards to a reduction in exacerbation rate with indacaterol monotherapy. No differences were reported in dyspnoea, health status or rescue medication use between treatment groups. During the trial, no patients in the indacaterol group reported pneumonia, compared with two patients in the SFC group. One patient in the indacaterol group reported pneumonia 5 days following completion of the study.

Authors conclude that patients with moderate airflow limitation and no exacerbation history can be withdrawn from SFC if they are switched to an effective long-acting bronchodilator, indacaterol, with no change in lung function, exacerbation rate and patient-reported outcomes.

### COSMIC

The COSMIC (COPD and Seretide: a Multi-centre Intervention and Characterisation) study explored the long-term effects of ICS withdrawal on exacerbations, lung function, symptoms and health status in patients with moderate-to-severe COPD (prebronchodilator FEV_1_ 30–70% predicted) and ⩾2 exacerbations in the previous year.^29^ Prior to study enrolment, 3%, 22% and 63% of patients on average were previously treated with LABA, ICS and LABA/ICS, respectively. Patients received 50/500 μg of SFC twice daily during a 3-month run-in period and then were randomised 1:1 (*N*=373) to continue receiving SFC or withdraw ICS to receive twice-daily salmeterol 50 μg alone for 52 weeks.

No significant difference was observed between the groups in the annual rate of moderate (requiring prescription of oral corticosteroids) or severe (hospitalisation) exacerbations when combined or considered separately, although the annual moderate-to-severe exacerbation rate was 1.2-fold greater in the ICS withdrawal group (95% CI 0.9 to 1.5; *P*=0.15). Rates of mild exacerbations (⩾3 extra inhalations of rescue medication per 24 h on ⩾2 consecutive days) were greater in the ICS withdrawal group compared with the ICS continuation group (adjusted relative rate 2.0; 95% CI 1.1, 3.5; *P*=0.016). However, the statistical model used in this analysis was not adjusted for between-patient variability, and hence the *P* values were underestimated (i.e., achieved greater statistical significance than might have been the case if between-patient variability had been accounted for).^30,31^ Patients with an FEV_1_ 30–49% and <30% predicted had higher rates of, and shorter time to, severe exacerbation compared with those with an FEV_1_ ⩾50% predicted, regardless of whether ICS treatment was withdrawn or continued.

Differences were seen between groups for several secondary outcomes. Patients in the ICS withdrawal group had fewer rescue medication-free days compared with the ICS continuation group (47% vs 53%; *P*=0.014). The ICS withdrawal group also had higher dyspnoea scores and numbers of disturbed nights’ sleep (*P*<0.001 for both). Mean FEV_1_ declined rapidly in the ICS withdrawal group in the first month, and then the decline stabilised to a similar rate to the ICS continuation group. After 12 months, FEV_1_ was significantly lower in the ICS withdrawal group than in the ICS continuation group (mean adjusted difference 4.1%; ~50 ml; *P*<0.001). No significant differences were reported in health status; however, the overall adjusted difference in Clinical COPD Questionnaire score between the two groups was statistically significant (0.13; *P*=0.041). Pneumonia rates were not reported.

The authors concluded that ICS withdrawal in patients with moderate-to-severe airflow limitation and frequent exacerbations leads to deterioration in lung function and dyspnoea, and an increased frequency of mild exacerbations. These findings support current treatment strategies recommending ICS treatment for patients at high risk for exacerbations.^1^ As high-risk (exacerbating) patients in the study should, according to management recommendations, have been receiving ICS-based treatment, this may not have been the most appropriate patient population in which to study ICS withdrawal.

### WISDOM

The WISDOM (Withdrawal of Inhaled Steroids During Optimised bronchodilator Management) study investigated the effects of stepwise withdrawal of ICS on exacerbation risk in patients with severe-to-very-severe COPD (FEV_1_<50% predicted) and at least one exacerbation in the previous 12 months.^32^ Prior to the study, 46.9%, 64.6% and 69.9% of patients were receiving long-acting muscarinic antagonists (LAMAs), LABAs or ICS, respectively, and 39.0% were receiving all three treatments combined. All patients received triple therapy of 18 μg of tiotropium once daily, 50 μg of salmeterol twice daily and 500 μg of FP twice daily for a 6-week run-in period. Patients were then randomised 1:1 (*N*=2,485) to either continue receiving triple therapy for 52 weeks or withdraw FP in three stages over the initial 12 weeks of the 52-week treatment period.

**Figure 3:**
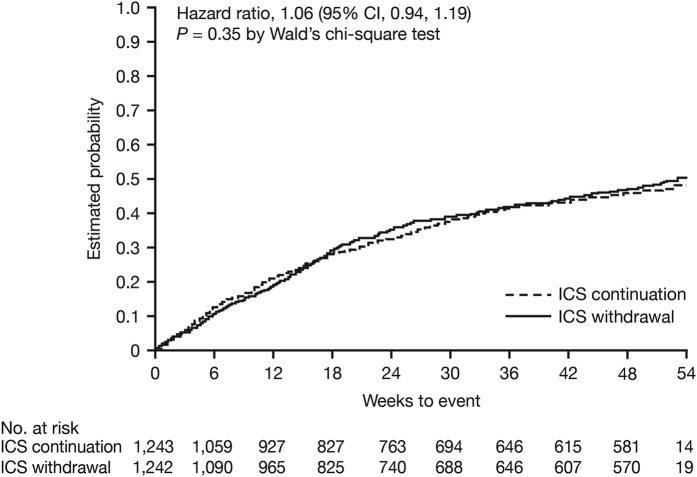
WISDOM: probability of moderate or severe exacerbations over 54 weeks in patients with severe-to-very-severe COPD (FEV_1_<50% predicted) and ⩾1 exacerbation in the year prior to screening.^32^ Hazard ratio is for ICS withdrawal versus ICS continuation. CI, confidence interval; ICS, inhaled corticosteroid. Reproduced from *New England Journal of Medicine*.^61^ Copyright © (2014) Massachusetts Medical Society. Reprinted with permission from Massachusetts Medical Society.

**Figure 4:**
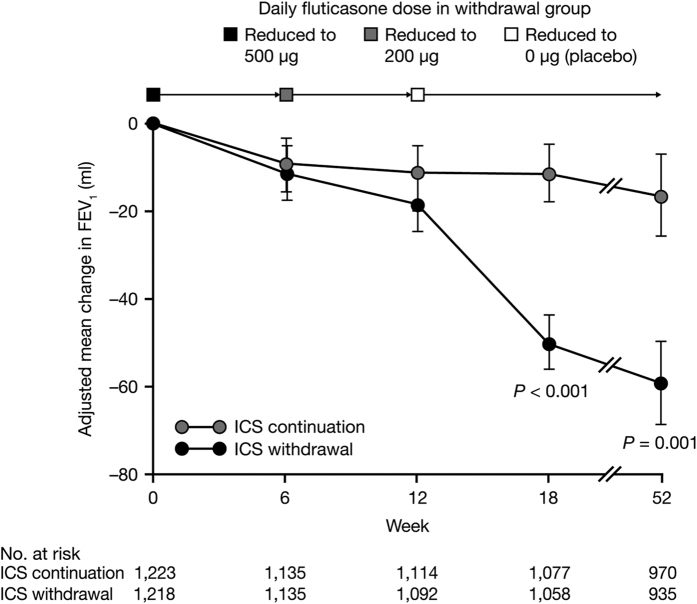
WISDOM: adjusted mean change in FEV_1_ in patients with severe-to-very-severe COPD (FEV_1_ <50% predicted) and ⩾1 exacerbation in the year prior to screening.^32^ Adjusted mean change in FEV_1_ during the 52-week study period in WISDOM. FEV_1_, forced expiratory volume in 1 s; ICS, inhaled corticosteroid. Reproduced from *New England Journal of Medicine*.^61^ Copyright © (2014) Massachusetts Medical Society. Reprinted with permission from Massachusetts Medical Society.

At 52 weeks, ICS withdrawal in a stepwise manner was non-inferior to ICS continuation with respect to the risk for moderate-to-severe exacerbations (HR 1.06; 95% CI 0.94, 1.19; Figure 3). The decline in dyspnoea (change from baseline in modified Medical Research Council score) did not differ significantly between the two groups (0.035 and −0.028 for ICS withdrawal and ICS continuation, respectively; *P*=0.06). St George’s Respiratory Questionnaire total score increased by 1.15 units in the ICS withdrawal group and decreased by 0.07 units in the ICS continuation group from baseline to week 52 (*P*=0.047). Following complete withdrawal of ICS at week 18, the mean decline in trough FEV_1_ was 38 ml greater in the ICS withdrawal group than in the ICS continuation group (*P*<0.001; Figure 4), with the difference remaining similar (43 ml) at week 52. There were no differences in rates of pneumonia over 52 weeks (5.5 and 5.8% in the ICS withdrawal group and ICS continuation group, respectively).

Concerns have been raised about the duration of WISDOM, and whether a 1-year period is long enough to capture exacerbations.^33^ In addition, the inclusion criteria did not limit enrolment to frequent exacerbators as defined by GOLD (⩾2 exacerbations per year or ⩾1 exacerbation with hospitalisation)^1^ and patients with previous mild events were eligible for inclusion; furthermore, many patients were not receiving ICS treatment prior to study entry, suggesting a low exacerbation history. Commenters also suggested that by excluding patients with no exacerbations the study does not address patients in whom ICS were having a preventative effect on exacerbations.^33,34^ It is important to note that, although patients were all receiving triple therapy in the 6-week run-in period, only 39% were receiving triple therapy prior to study entry.^32^ Therefore, treatment was stepped up for many patients in run-in ahead of ICS withdrawal, which may have affected the outcomes.

Within this context of these potential limitations, WISDOM provides information on stepwise withdrawal of ICS to LABA plus LAMA treatment in patients with severe but stable COPD. Results indicated that ICS withdrawal was not associated with an increase in the risk for moderate-to-severe exacerbations compared with continued ICS treatment, and after an initial significant decline following withdrawal, lung function declined at a similar rate with ICS withdrawal and ICS continuation. Improvement in dyspnoea did not differ significantly between the two groups, and the difference in health status did not reach clinical importance.

### Observational studies

The OPTIMO (Real-Life study On the aPpropriaTeness of treatment In MOderate COPD patients) study was not retrieved in our literature search; however, it is included here as we consider it of relevance to discussions on ICS withdrawal.^35^ The objective of this multicentre, prospective, real-life study was to explore whether ICS withdrawal in patients with an FEV_1_>50% predicted and <2 exacerbations per year was associated with a decline in lung function, deterioration in symptoms and an increase in exacerbation rate. Patients receiving ICS/LABA (either as a fixed-dose combination or via separate inhalers) were recruited into the study, and a decision regarding whether to maintain or withdraw ICS treatment was made by their physicians, who had been adequately informed on the content of the GOLD strategy document in the start-up meeting of the study. Where ICS treatment was withdrawn, patients were predominantly switched to long-acting bronchodilator monotherapy (tiotropium, indacaterol, formoterol or salmeterol), or combined bronchodilator treatment (tiotropium plus indacaterol). The remainder of patients switched to short-acting bronchodilators and/or theophylline. Of the 816 patients who completed the study, 482 continued with ICS/LABA treatment, whereas 334 patients had their ICS component withdrawn. At the end of the 6-month observational period, there was no significant difference in FEV_1_% predicted, COPD Assessment Test scores or number of exacerbations (defined as a change in symptoms leading to a brief course of antibiotics, systemic corticosteroids or both) between patients who changed their treatment (ICS withdrawal group) and those continuing on ICS/LABA combinations. Although the authors acknowledged that lack of randomisation was a major limitation of this study, they concluded that OPTIMO provides observational evidence that in patients with moderate airflow limitation and infrequent exacerbations (<2 per year) ICS can be withdrawn without increasing the risk of exacerbations, provided adequate bronchodilator treatment is in place.

## Discussion

ICS use is common among patients diagnosed with COPD, with limited conformity to global recommendations or clinical trial data.^1,15^ The benefits of ICS treatment over placebo were demonstrated in early studies.^36–39^ However, these studies failed to consider the benefits of maximal bronchodilation with long-acting bronchodilator therapy in exacerbation prevention, and their conclusions may therefore be judged to be of little value in the current therapeutic environment. Analyses of active comparator studies demonstrate that ICS have additional benefit to LABAs when used in combination in some patient populations (including those with asthma-COPD overlap syndrome or severe COPD), but not in others.^19,40,41^ Currently, many patients considered to be at low risk for exacerbations are receiving ICS^7–14^ in spite of recommendations to the contrary.^1^ Accordingly, we believe that ICS withdrawal studies provide important information for directing future clinical decisions for patients unlikely to benefit from ICS.

Trials in which patients are withdrawn from ICS to placebo provide limited relevant evidence for current clinical practice, as all COPD guidelines recommend ICS only as an add-on treatment to long-acting bronchodilator therapy.^1,4,42^ Although both of the placebo-controlled trials allowed some use of bronchodilator therapy, neither used therapy consistent with current recommendations. Therefore, we focus the discussion of this review on the evidence from active comparator trials. For clinical clarity, we discuss the effects of ICS withdrawal in low-risk and high-risk patients separately.

### Low-risk patients

The INSTEAD patient population most closely exemplifies low-risk patients who should not be receiving ICS based on current recommendations, with enrolment in the study limited to those with moderate COPD (FEV_1_ 50–80% predicted) and no exacerbations in the previous 12 months.^28^ In this patient population, withdrawal of ICS (which all patients had taken for at least 3 months) had no adverse effect on lung function, exacerbations or patient-reported outcomes.^28^ Observational data from the real-life prospective OPTIMO study, which also studied low-risk patients, substantiate these findings.^35^ Although not an ICS withdrawal study, the ILLUMINATE trial did enrol and randomise low-risk patients (moderate COPD and no exacerbations in the previous year) to either the LABA/LAMA combination indacaterol/glycopyrronium 110/50 μg once daily or SFC 50/500 μg twice daily.^43^ In the group randomised to indacaterol/glycopyrronium, 30% of patients were withdrawn from previously prescribed ICS during the washout phase.^43^ In subanalyses of those 30%, no differences were seen in outcomes.^43,44^ Specifically, lung function was improved with indacaterol/glycopyrronium at study end versus SFC, regardless of ICS withdrawal.^43^ We conclude, therefore, that patients with moderate COPD and no exacerbations in the previous year are appropriate candidates for ICS withdrawal, if adequate bronchodilation is in place.

### High-risk patients

Recent data suggest that the use of LABAs and LAMAs in combination may also prevent exacerbations in patients with severe-to-very-severe COPD and a history of exacerbations,^45^ making ICS withdrawal studies in high-risk patients of potential clinical value.

In COSMIC, withdrawal of ICS did not significantly affect the rate of moderate-to-severe or all exacerbations in patients classed as high risk (however, it is important to note that the *P* values were underestimated due to the statistical model).^29^ In addition, in WISDOM the risk for moderate-to-severe exacerbation was similar with ICS withdrawal and continued ICS treatment,^32^ suggesting that ICS withdrawal did not affect more severe exacerbations in this patient population. The rate of moderate-to-severe exacerbations was assessed as a secondary end point in both trials, and both trials only assessed exacerbation risk over a period of 52 weeks, which critics indicate may not be long enough to assess differences following ICS withdrawal.^46^ In addition, COSMIC assessed withdrawal of ICS to salmeterol. We believe that further research is required regarding ICS withdrawal using newer, more effective LABAs as comparators.

COSMIC was not powered to compare exacerbation rates as a primary outcome and may have underestimated unreported or self-managed exacerbations; however, this appears unlikely to have affected the overall conclusions.^29^ In our view, unreported or self-managed exacerbations are most likely to fall into the mild exacerbation category, already noted to be more common in the ICS withdrawal group than in the ICS continuation group.^29^ However, mild exacerbations did not appear to deteriorate into moderate or severe exacerbations.^29^ The WISDOM trial study design may also be criticised, as this was not a withdrawal study but a treatment enhancement study for some patients in whom ICS was added during the ‘run-in’ period. However, it should be noted that this enhanced therapy group was small.^32^

In addition to data from the withdrawal trials, in the 2-year INSPIRE (Investigating New Standards for Prophylaxis in Reducing Exacerbations) study, there was no difference in exacerbation rate with SFC and tiotropium in high-risk patients.^47^

A small but significant deterioration in lung function (~50 ml) was observed among those withdrawn from ICS versus those continuing ICS treatment in both COSMIC and WISDOM.^29,32^ The mechanism and clinical significance of this deterioration are currently unknown. In the TORCH study, there was an increase in FEV_1_ with LABA/ICS compared with LABA monotherapy, suggesting that the addition of ICS had a small bronchodilatory effect.^48^ It is possible that withdrawal of ICS from LABA/ICS treatment may result in an immediate loss of the synergistic effect between the ICS and β_2_-agonist,^49–51^ leading to an immediate drop in FEV_1_. Following this initial reduction, lung function decline may then stabilise. Following the immediate deterioration in lung function in the ICS withdrawal group in COSMIC and WISDOM, the trajectory of lung function decline resembled that seen in the ICS continuation group (Figure 4).^29,32^ Similarly in TORCH (although not a withdrawal study), decline in FEV_1_ was similar between SFC and the monocomponents between weeks 24 and 156 (−39 ml for SFC and −42 ml for both fluticasone and salmeterol).^48^ It is important to note that a 1-year study may not be sufficient to make conclusions about declining lung function following withdrawal, nor can the data be extrapolated to longer time periods. More long-term studies (at least 3–4 years in length) into the impact of ICS withdrawal on lung function decline are required.

Further studies are needed into the effects of ICS withdrawal in high-risk patients before any conclusions may be drawn. Indeed, we believe that further analyses of different subgroups or phenotypes of those at high risk of exacerbation should be completed. For example, in the COSMIC population, patients with severe-to-very-severe COPD were at much higher risk of an exacerbation following ICS withdrawal compare with those with moderate COPD despite all groups having a history of two or more exacerbations in the previous year.^29^ Further analyses are also required in high-risk patients into the efficacy of dual bronchodilators compared with LABA/ICS with regard to exacerbation prevention. Ongoing studies such as FLAME (NCT01782326) may provide data for this comparison.^52^

### Safety

The safety risks associated with ICS use, such as pneumonia, have been reviewed extensively.^18–23^ Mechanistically, high local concentrations of ICS in the lungs may increase the risk of pneumonia owing to immunosuppressive effects,^53,54^ or through inhibition of NF-κB.^55^ The European Medicines Agency (EMA) is currently investigating the pneumonia risk associated with ICS use in COPD, and the potential need to revise existing prescribing advice.^56^ ICS-associated pneumonia risk was previously investigated by the EMA in 2010; however, given that further evidence is now available, the EMA consider it necessary to perform a thorough review and have requested information from market authorisation holders regarding pneumonia risk associated with ICS-containing products.^57^

The impact of ICS on pneumonia risk is apparent within the first year of use, where it peaks and then remains both elevated and stable over long periods of continuous use.^20^ Data suggest that risk for pneumonia is significantly reduced following ICS withdrawal, which may further support ICS withdrawal in patients who are unlikely to benefit, or those who are at risk for pneumonia.^20,58^ Pneumonia rates were not reported in the majority of the withdrawal trials discussed. The 1-year WISDOM study reported no difference in pneumonia risk between triple therapy and ICS withdrawal arms.^32^ However, pneumonia rates decline gradually following withdrawal, where it can take at least 4 months for the risk to fall.^20^ In a population-based observational study, pneumonia risk was reduced by 20% by the first month after ICS withdrawal, 50% by the fourth month, at which point it stabilised ~50%.^58^ The stepwise withdrawal of ICS over 3 months in WISDOM may have had a longer-term effect that could not be observed during the limited follow-up of that trial, thus masking the effect of ICS withdrawal on pneumonia risk.

## Conclusions

Data from the OPTIMO observational study and the INSTEAD randomised trial are consistent in demonstrating no clinically significant effect of ICS withdrawal up to 6 months in patients at low risk for exacerbation when sufficient bronchodilation is in place. Several other trials have shown limited additional benefit of ICS beyond that provided by bronchodilators. In our opinion, these data support the limited use of ICS and potential withdrawal of ICS in the management of low-risk patients with COPD. The findings from OPTIMO are also recognised by GOLD as representative of the safety of ICS withdrawal in low-risk patients, provided they are left on maintenance treatment with long-acting bronchodilators.^1^ Withdrawal of ICS in those with severe COPD requires continued study, further to COSMIC and WISDOM, but appears to be worth additional consideration. We conclude that clinical decisions in the management of patients with moderate COPD should include withdrawal of ICS treatment in many of those for whom it is currently prescribed.

## Methods

Relevant medical literature on ICS withdrawal was identified by searching the PubMed (Medline) database for articles published in English before January 2015, limited to destinations of ‘randomised controlled trials’ or ‘clinical trial’. Search terms included the following: ‘chronic obstructive pulmonary disease’ OR ‘COPD’ AND ‘inhaled corticosteroids’ OR ‘ICS’ OR ‘glucocorticoids’, AND ‘withdrawal’, OR ‘switch’. We also manually examined bibliographies from publications identified through the initial searches for further relevant literature. We focused on peer-reviewed, published manuscripts of ICS withdrawal trials that examined clinical end points associated with the treatment of COPD. No meta-analyses were attempted.

## Figures and Tables

**Table 1:** Overview of trials involving withdrawal of ICS in COPD, grouped by treatment comparators and disease severity

*Design*	*Patient population*	N	*Treatment groups*	*Duration of ICS use*	*Withdrawal*	*Outcomes*
*Placebo-controlled trials*						
COPE (van der Valk, P *et al.*^25^)						
6-month, randomised, double-blind, parallel-group study	• Moderate-to-severe COPD (prebronchodilator FEV_1_ 25–80% predicted) • No exacerbations in the month prior to enrolment	244	• FP 500 μg b.i.d. • Placebo	FP for 4-month run-in period	Abrupt, on randomisation	Earlier exacerbation with placebo versus FP (HR 1.5; 95% CI 1.05, 2.1)
WISP (Choudhury AB *et al.*^27^)						
52-week, randomised, double-blind, placebo-controlled, parallel-group	• Moderate-to-very-severe COPD (FEV_1_ <80% predicted)	260	• FP 500 μg b.i.d. • Placebo	Median 8 years (prior to study entry)	Usual ICS stopped on study entry, and FP or placebo started	Increased exacerbation risk with placebo versus FP (RR 1.48; 95% CI 1.17, 1.86; *P*<0.001)
						
*Active comparator trials*						
INSTEAD (Rossi A *et al.*^28^)						
26-week, randomised, double-blind, double-dummy, parallel-group study	• Moderate COPD (FEV_1_ 50–80% predicted) • No exacerbations for >1 year prior to study entry	581	• SFC 50/500 μg b.i.d. • Indacaterol 150 μg q.d.	SFC for ⩾3 months	Abrupt, on randomisation	Non-inferiority of indacaterol to SFC in trough FEV_1_ after 12 weeks (mean treatment difference −9 ml; 95% CI −45, 26)
COSMIC (Wouters EF *et al.*^29^)						
52-week, randomised, double-blind, parallel-group study	• Moderate-to-severe COPD (FEV_1_ 30–70% predicted) • ⩾2 exacerbations in previous year	373	• SFC 50/500 μg b.i.d. • Salmeterol 50 μg b.i.d.	SFC for 3-month run-in period	Abrupt, on randomisation	Greater decline in FEV_1_ with salmeterol versus SFC (4.1%; 95% CI 1.6, 6.6; *P*<0.001)
WISDOM (Magnussen H *et al.*^32^)						
52-week, randomised, double-blind, parallel-group, active-controlled study	• Severe-to-very-severe COPD (FEV_1_<50% predicted) • 1 exacerbation in the year prior to screening	2,485	• Tiotropium 18 μg q.d.+salmeterol 50 μg b.i.d.+FP 500 μg b.i.d. • Tiotropium 18 μg q.d.+salmeterol 50 μg b.i.d.	Triple therapy for 6-week run-in period	Stepwise reduction in FP dose every 6 weeks	Non-inferiority of ICS withdrawal to ICS continuation in time to first moderate or severe exacerbation (HR 1.06; 95% CI 0.94, 1.19)

Abbreviations: b.i.d., twice daily; COPD, chronic obstructive pulmonary disease; COPE, COPD study of the department of Pulmonary Medicine, Enschede; CI, confidence interval; FEV_1_, forced expiratory volume in 1 s; FP, fluticasone propionate; HR, hazard ratio; ICS, inhaled corticosteroid; q.d., once daily; q.i.d., four times daily; RR, relative risk; SFC, salmeterol/fluticasone propionate combination.
